# Engaging communities as partners in health crisis response: a realist-informed scoping review for research and policy

**DOI:** 10.1186/s12961-024-01139-1

**Published:** 2024-05-06

**Authors:** Mateus Kambale Sahani, Harro Maat, Dina Balabanova, Mirkuzie Woldie, Paul Richards, PARES Research Group, Lawrence S Babawo, Negalign Berhanu, Sander Koenraadt, Diribe Makonene, Susannah H Mayhew, Vikas Mohan, Esther Mokuwa, Justine Namakula, Edith Ngunjiri, Freddie Ssengooba, Hakimu Sseviiri, Revocatus Twinomuhangi, Ahmed Vandi, Susannah Mayhew

**Affiliations:** 1 Department of Global Health and Development, Faculty of Public Health and Policy, London School of Hygiene and Tropical Medicine, London, UK; 2 Knowledge, Technology, and Innovation Group, Department of Social Sciences, Wageningen University, Wageningen, The Netherlands; 3 Department of Health Policy and Management, Jimma University, Jimma, Ethiopia; 4 School of Environmental Sciences, Njala University, Freetown, Sierra Leone

**Keywords:** Community Engagement, Community participation, Crisis response, Realist review, Scoping review, Outbreak, Emergency, Resilience, Partnership, Collaboration

## Abstract

**Background:**

Health is increasingly affected by multiple types of crises. Community engagement is recognised as being a critical element in successful crisis response, and a number of conceptual frameworks and global guideline documents have been produced. However, little is known about the usefulness of such documents and whether they contain sufficient information to guide effective community engagement in crisis response. We undertake a scoping review to examine the usefulness of conceptual literature and official guidelines on community engagement in crisis response using a realist-informed analysis [exploring contexts, mechanisms, and outcomes(CMOs)]. Specifically, we assess the extent to which sufficient detail is provided on specific health crisis contexts, the range of mechanisms (actions) that are developed and employed to engage communities in crisis response and the outcomes achieved. We also consider the extent of analysis of interactions between the mechanisms and contexts which can explain whether successful outcomes are achieved or not.

**Scope and findings:**

We retained 30 documents from a total of 10,780 initially identified. Our analysis found that available evidence on context, mechanism and outcomes on community engagement in crisis response, or some of their elements, was promising, but few documents provided details on all three and even fewer were able to show evidence of the interactions between these categories, thus leaving gaps in understanding how to successfully engage communities in crisis response to secure impactful outcomes. There is evidence that involving community members in all the steps of response increases community resilience and helps to build trust. Consistent communication with the communities in time of crisis is the key for effective responses and helps to improve health indicators by avoiding preventable deaths.

**Conclusions:**

Our analysis confirms the complexity of successful community engagement and the need for strategies that help to deal with this complexity to achieve good health outcomes. Further primary research is needed to answer questions of how and why specific mechanisms, in particular contexts, can lead to positive outcomes, including what works and what does not work and how to measure these processes.

## Introduction

People around the world are increasingly experiencing varied and multiple health shocks which have a critical impact on people’s life, livelihoods and wellbeing [1–4]. Climate change and environmental degradation (often exacerbated by human activity) have been shown to be linked to outbreaks of zoonotic diseases [5–12]. Collectively, and in combination, these challenges lead to serious and ongoing uncertainty for many populations who face the prospect of ongoing poverty and multiple health shocks, such as deadly epidemic, floods and landslides [3, 13–18].

When faced with a health-related shock or crisis, the first responders are usually those who are immediately affected, including both community members and local frontline health staff [16–21]. Because community members often have detailed practical knowledge about their environment, they are also often effective first-responders and play a critical role in creating viable and effective solutions to health crises/shocks [3, 13, 16–18, 21–23].

Researchers have shown the importance of engaging frontline personnel to effectively respond to a crisis [1–4]. There is evidence showing that engaging local communities in the response is an effective way to positively respond to health emergencies [13, 15–18, 20, 22]. These studies emphasize that a community should be taken as a genuine partner and involved at all levels of the response starting from initial planning [13–15]. Most importantly, this partnership should be established well ahead of the crisis to build trust and ownership [3, 13, 15, 16, 18, 20]. There is diversity in documented approaches used to engage communities in responding to health shocks across different contexts and types of communities. Some responders have used ‘science shops’, a type of citizen science by which community members participate in formulating research questions and co-design (experimental) interventions to produce solutions to a crisis [43–45]. Other citizen science projects ensure that community members own data production and products that can be used within the local conditions [43, 45]. These approaches have shown positive effects in the agricultural sector for response to drought and food production crises [5, 43, 45]. However, the evidence base – particularly in the health sector – is slim and there is little understanding of what engagement actions work and in what contexts, what positive impacts they can produce or why some communities appear to be more resilient than others [3, 4, 13–22, 24–26].

Taking community involvement in crisis response seriously requires understanding local knowledge and how it translates into practice and embedding this into guidance documents and crisis response implementation protocols. Since the Ebola epidemics in West Africa in 2013–2015 and the global coronavirus disease 2019 (COVID-19) pandemic a plethora of guidance and literature on ‘community engagement’ in responding to outbreaks have emerged, some updated to African region. No published review of these frameworks or global guideline documents currently exists, which synthesises their insights and examines their usefulness for those seeking to implement or evaluate community engagement initiatives.

There is evidence that engaging the community in the problem-solving process is key to effective responses and stimulates the sense of ownership bringing the community on board in seeking for solution of the health crisis even though this is still not always recognised and not implemented [27]. Researchers found that vulnerable populations, such as refugees and internally displaced people, are prone to risk of frequent outbreak owing to shortage or poor quality of water and lack of access to healthcare [28]. Coordination between civil society organisations (CSO) and health authorities is key to support people in need, particularly marginalized groups [27, 28]. It was shown that involving the community in the design and implementation of the crisis response is crucial for success and control of pandemics [29].

Prompt and immediate communication to the community members during health crisis helps to avoid false information and misunderstanding leading to positive outcome and health improvement [30].

Bringing rich insights from a realist-informed lens, this study reviews the ways in which community involvement and engagement is defined and used in relevant formal guideline documents, specifically in crisis response, and in theoretical and conceptual articles seeking to define the concepts. In particular, our paper examines the extent to which sufficient detail is provided on the contexts, mechanisms and outcomes of an effective community involvement. Additionally, we examine whether it is possible to discern – and therefore act on – the interactions between these elements and identify gaps and limitations in existing frameworks and guidance. This is a necessary first step to improving research, policy and practice on this critical topic.

## Methods

Providing meaningful analysis of global guidelines and frameworks on effective community involvement mechanisms and processes in complex crisis settings is not well served by the accepted systematic review methods, therefore we employ a scoping review informed by realist review methods. Realist review is a method for studying complex interventions in response to the perceived limitations of conventional systematic review methodology [31, 32]. It involves identification of contexts, mechanisms and outcomes (CMOs) of interventions or actions to understand what community engagement interventions work or do not work when applied in different contexts or circumstances and deployed by different stakeholders [31, 32].

### Scope and search strategy

Studies were identified and data extracted and aggregated systematically following a realist-informed approach, from May 2022 to July 2023, which was the period for literature screening and data extraction. This involved an iterative scoping of available literature and documents to understand the contexts in which specific community engagement mechanisms can lead to positive outcomes for improving responses to public health emergencies.

The databases searched were Google Scholar, PubMed and Global Health. In addition, we identified scholars with particular expertise in on community engagement in crisis response who recommended additional sources (articles and books). The literature search was systematic using the following key terms: Community participation OR Community resilience OR community sustainability OR community persistence OR Community involvement OR Community engagement OR health crises OR Community preparedness AND public health emergencies. In the advanced search model, the following sentence was added in addition to the above key words: ‘community participation in responding to health crises’. The study covered documents published or made available (for grey literature) in the period between January 1995 to July 2023.

The initial database searches produced a large number of papers, which included conceptual papers and case studies. We were also interested in practical global guidelines documents as well as reports of interventions from grey literature sources. For reasons of practicality, we separated out our analyses of case study literature (not reported here) from our analysis of conceptual literature and formal guideline documents (grey literature) which is the focus of this article; the gap in understanding the gaps in the latter is particularly critical as these often inform crises responses by international agencies and governments. We report on two themes. First, we analyse the theoretical and conceptual literature on community engagement in crisis response to understand whether and how published papers in this field conceptualise the contexts (C) in which mechanisms (M) of community engagement can lead to positive outcomes (O) in a health crisis. Second, we analyse the grey literature from international agencies and non-Governmental Organizations (NGOs), such as the United Nations Children’s Fund (UNICEF), World Health Organization (WHO), GOAL, International Rescue Committee (IRC), International Federation of Red Cross and Red Crescent Societies (IFRC) and Global Outbreak Alert and Response Network (GOARN), which provides official guidance on how to conduct community engagement, to understand whether these documents consider the different contexts of the interventions (mechanisms) they propose and whether they clearly identify how mechanisms lead to outcomes. Lessons from both of these types of sources can inform effective community engagement actions.

The selection criteria for the source documents were discussed in advance in several meetings with the research team. All the documents included in the review were screened against this set of criteria, as shown on the PRISMA chart below.

**Fig. 1:**
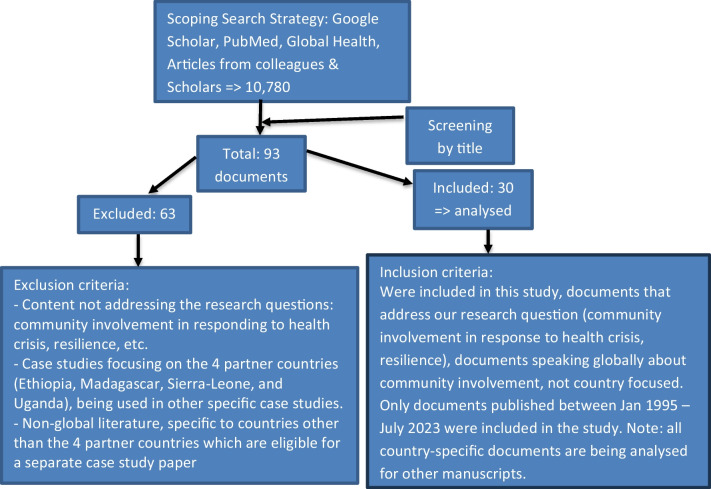
Literature search and selection strategy

The initial search produced 10,780 documents published from January 1995 to July 2023 which were searched with key words and manual scanning of titles. For this paper (conceptual frameworks and guideline documents from organisations, such as the WHO, UNICEF, OXFAM, GOAL, ICRC and IFRCRC), a total of 93 documents were found to be relevant. The abstracts of these 93 documents were then screened using a set of inclusion and exclusion criteria shown in the PRISMA chart below. After title and abstract screening, 63 documents were excluded, and 30 documents were included in the full-text analysis reported in this paper (Fig. 1).

### Theoretical framework for analysis

**Fig. 2:**
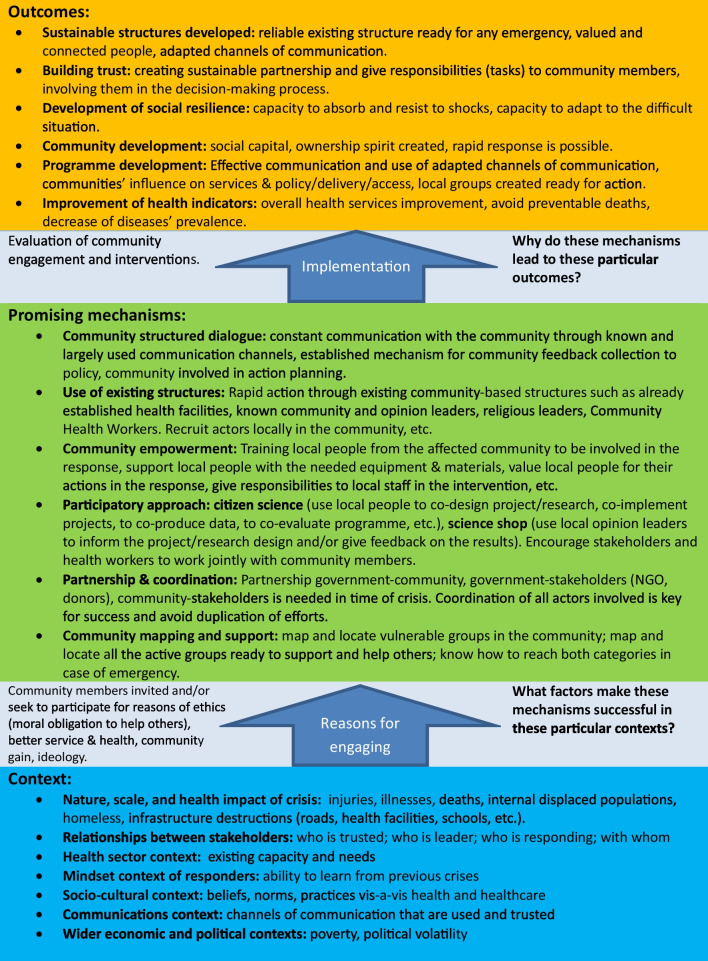
Framework of key context-mechanism-outcome considerations

As noted above, our analysis was informed by the realist review approach. However, we have found that this was not entirely adequate for our literature review study, which required more specific details and parameters in relation to community engagement. Hence, we combined it with details from Brunton’s framework on community engagement [16] which considers the reasons why people engage in local-level actions (which aligns to the context domain in a realist analysis), the dimensions and models of engagement (mechanisms domain), and what sorts of outcomes (personal, community, health etc. – outcome domain) this leads to. Our combined framework (Fig. 2 seen in the discussion) was developed inductively after examining how existing frameworks and global guidelines on community engagement in crisis response described: the specific local health crisis contexts to which communities were responding which could shape engagement mechanisms; the range of mechanisms (actions/processes) that were developed and employed to engage communities in crisis response; and what outcomes these mechanisms achieved in specific contexts (from an increase in participation of community members in decision making or implementation activities through observable impacts on reducing infectious disease spread).

Data were extracted from literature using an Excel template to organise the data, by literature source and type (conceptual paper or guideline document), according to the information they contained on context, mechanisms, and outcomes (CMOs) relating to community engagement in crisis response. Regarding context, we looked for information on how it was defined (C) in terms of the community engagement programme and key issues that are related to global health crisis, such as epidemic/outbreak, or the combination of multiple events (syndemics). Under mechanisms, we identified what range of specific community engagement activities, interventions, processes or strategies were implemented (M) and reported. The detailed information sought were related to the following promising mechanisms: community dialogues, empowerment, the use of existing resources in the community, partnerships, mapping and inclusion of vulnerable groups. For outcomes, we looked for information on how outcomes were defined and which ones were seen as important to achieve (O); they were related to population health, programme and community development and sustainability of community structures. We then examined the extent to which the documents were able to articulate how contexts, mechanisms and outcomes (CMOs) interacted and whether they were able to define how, why and under what circumstances particular outcomes could be achieved using specific mechanisms. By interaction, we mean a linkage between context and mechanism and between mechanism and outcome.

During the analysis it became clear that not all included documents contained information on all CMO categories (context, mechanisms and outcomes) and sometimes there was insufficient information. Each document was reviewed by reading and extracting relevant text under each CMO category into the Excel data extraction template. When a document provided some information in a particular CMO category but with too little detail to enable a good understanding, the document was recorded as containing ‘partial’ information about that category. When a document provided sufficient detail to enable a good understanding of the CMO category in relation to community engagement, then it was recorded as ‘Y’ (i.e. ‘yes, information is contained’) and where it contained no information it was recorded as ‘N’ (i.e. ‘no information’).

### Ethical considerations

As a literature review, this study did not require ethics approval, however it was conducted as part of a wider project on community engagement in crisis response, named Partnerships for Resilience (PARES), which has received ethics approval from a range of implementing countries (not relevant to this review) and the London School of Hygiene & Tropical Medicine (relevant to this review): Ethics Review Committee Approval Number 29783.

## Results

### Description of documents

Thirty documents were included in our analysis; 21 of these were papers that developed conceptual or theoretical frameworks on community engagement and responsiveness in crisis response and 9 were guideline documents on community engagement in crisis settings prepared by agencies and organisations operating globally.

The theoretical and conceptual papers were based on reviews of literature across multiple settings, a combination of review and personal observations or experiences or experiences of experts involved in emergency response. These documents were generally written by experts or staff within those organisations, sometimes in collaboration with external consultants, though document authors were not always disclosed. Table 1 shows the range of reviewed documents in terms of whether they considered the context, mechanisms and outcomes of community engagement activities.

**Table 1:** Included information on context, mechanism, outcome and interactions, in the reviewed documents

Source (and reference #)	CMO information included (Y/N)			
	Context	Mechanism	Outcome	Interactions
Section 1. Theoretical and conceptual literature				
Marston, Renedo and Miles, 2020 [18]	Partial	Partial	Partial	N
Abimbola and Topp, 2018 [37]	Y	Partial	N	N
Schoch-Spana, Franco, Nunzo and Usenza, 2007 [33]	Partial	Partial	Y	N
Turoff and Van de Walle, 2004 [34]	Partial	Partial	Y	N
Heath & Palenchar, 2000 [35]	N	Y	Y	N
Dynes, 1997 [25]	N	Y	Y	N
van de Gevel, van Etten and Deterding, 2020 [49]	N	Y	Y	N
Steinke, van Etten and Pablo MZ, 2017 [51]	N	Y	Y	N
Herzog and Lepenies, 2022 [50]	N	Y	Y	N
Madrigano, Chandra et al., 2017 [52]	N	Y	Y	N
Nuzzo and Meyer, et al., 2019 [36]	Y	Y	N	N
Moya and Goenechea, 2022 [53]	Y	Partial	Y	N
Osterholm, Moore, Ostrowsky, Kimball-Baker and Farrar, 2016 [40]	Partial	Y	Y	N
Bhandari and Alonge, 2020 [38]	Y	Y	Y	Partial
Mayhew and Kyamusugulwa, et al., 2021 [4]	Y	Y	Y	Y
Ebola Gbalo Research Group, 2019 [1]	Y	Y	Y	Y
Manoncourt, Obregon and Chitnis, 2022 [26]	Y	Y	Y	Y
Maddah D., et al., 2022 [27]	Y	Partial	Y	Y
Bain L.E., et al., 2023 [28]	Y	Partial	N	N
Sahoo K.C., et al., 2023 [29]	Y	Y	Partial	N
Li K., et al., 2022 [30]	Y	Y	Partial	N
Section 2. Global guideline documents				
International Federation of Red Cross (IFRC), 2022 [46]	Partial	Y	N	N
McCrossan and Owen (GOAL), 2022 [20]	N	Y	Y	N
GOARN, UNICEFF, IFRC and WHO, 2020 [42]	N	Y	Y	N
IRC, 2021 [47]	Partial	Y	Partial	N
WHO, 2021 [41]	Partial	Y (detailed)	Partial	N
WHO, 2018 [44]	Y	Y	Partial	N
MacKay, Colangeli and Jaworski, et al., 2022 [43]	Y	Y	Y	N
WHO, 2021 [48]	Y	Y	Y	Partial
GOARN, 2022 [45]	Y	Y	Y	Partial

As can be seen, few documents were able to provide detailed information across all three CMO categories. Four conceptual papers do so, and three of these provided detailed analyses of Ebola outbreaks to inform their conceptual understanding. For example, Ebola Gbalo paper [1] includes a detailed description of the village context in which communities were involved in shaping responses to Ebola, and also contains significant detail on specific mechanisms and how these led to particular outcomes, so it is recorded as ‘detailed’ for those categories. The Manoncourt et al. paper [26] builds on the Ebola Gbalo paper to provide even greater detail on the West Africa Ebola response and is also noted as ‘detailed’ across all categories. The Mayhew et al. paper [4] provides similar levels of detailed analysis of the Ebola response in DR Congo that helped to develop a conceptual framework that envisages community engagement at multiple levels of crisis response. All three papers provided convincing descriptions of how and why the contexts of crisis response shaped community engagement mechanisms to achieve particular outcomes and are therefore rated ‘Y’ in the interactions column of the table. The fourth article by Bhandari and Alonge [33] is concerned with conceptualizing community resilience rather than community engagement per se, nevertheless it does offer a lot of useful detail on a range of engagement processes and actions in specific contexts that lead to particular outcomes. It is assessed as ‘Partial’ in its analysis of how and why interactions between contexts and mechanisms lead to certain outcomes for community engagement because this was not the main focus of the paper and information on the interactions specific to community engagement are often not explicit.

Among the global guideline documents, only three were able to provide details on all CMO categories and two of those sought to discuss the interactions between them, with the interaction analysis rated as ‘partial’ because it is not explicit or detailed. The WHO 2021 document [35] provides a range of useful examples of how different community engagement strategies were developed in different contexts and with what the outcomes were, though its analysis of the how and why is not very strong. The GOARN 2022 document [39] provides excellent analysis on the contexts and mechanisms possible for community engagement, grounded in concrete case examples. Outcomes are discussed in relation to what was achieved, with some analysis of how the interactions between them result in these outcomes in particular settings, for particular groups. The MacKay et al. [37] Social Media Guide is a very different document, focused on how to develop, implement and monitor crisis communication for public health using social media. Since it was not concerned with community engagement per se, it cannot provide useful analysis of CMO interactions for community engagement, nevertheless it does provide insightful information on each of the CMO categories that are relevant for involving communities in risk communication (though this is only a part of wider community engagement in crisis response).

### Information on context

#### Do theoretical and conceptual papers contextualise the response interventions?

Understanding the context in which each health crisis (shock) happens is crucial for successful implementation of response actions. Typically, ‘context’ is defined as the populations affected and their particular vulnerabilities [28]. The papers reviewed, however, revealed a need for wider understanding of the following aspects. First, the range of stakeholders available who could be expected to respond to the crisis and the nature of relationships and trust between them. Second, the capacity and needs of the health sector to respond. Third, the ability of responders to learn lessons from one emergency to the next.

There is recognition that the scale of health issues caused by natural disasters and epidemics and the diversity of the people they affect (different groups may be affected differently) require the engagement of many actors, not only those who serve as officials [33]. This in turn requires good coordination of actors for effective communication, ideally with suitable and adapted technologies that allow the right information to reach the community[34]. Several papers noted that good relationships and trust between communities and responders were critical to creating a context in which communication and engagement could be successful [16, 30–32]. As a prerequisite, several authors emphasised the need to understand how different communities define disaster and what their own coping mechanisms are which could then be supported and built upon rather than setting up conflicting or parallel mechanisms [16, 25, 30, 32]. For instance, communities played a key role in the COVID-19 response in the UK by complying with lockdowns and respecting other prevention measures [19]. This was possible because in the UK, as seen elsewhere, there was a supportive context in which citizens helped each other by checking on the wellbeing in different groups during the lockdown period, often organized through existing social networks [18, 25, 35].

The health system capacity to respond to health shocks was an important contextual element in many papers [36–38]. Weak health systems and lack of preparedness increase vulnerability to the health impacts of disasters, including infectious disease. In West Africa the local health system responded quickly and vigorously once the threat was recognized, but it could not provide adequate care to early affected populations when it had no resources to do so [personal protective equipment (PPE) and quarantine facilities were lacking in the early stages] [1, 26, 37, 39]. Exacerbating the existing limitations in health system capacities, the delays in international mobilization of support for testing, reporting and case identification for pandemic outbreaks reflected a macro-political context of weakness of leadership in international agencies for rapid intervention. The social consequences continue to be felt long after the outbreak [40].

An institutional context of lesson-learning among responders was seen as important in several papers. Many lessons are learned during previous experiences of crisis response, yet there is often a failure to act on them in future crises. The lessons from West Africa on the importance of building trust-based partnerships between affected communities and health crisis responders were ignored in the DRC leading to higher case fatality rates there [1, 4]. In addition, lessons from Africa could have been applied to western Covid-19 responses on how to mobilise and sustain communities as partners in the response but this was limited by the lack of existing mechanisms for knowledge exchange [18, 41].

#### Do global guideline documents consider contextualisation of the response?

In global guideline documents, the focus was on contextualising communication efforts in particular, rather than broader community engagement strategies. The need to assess the context in which communication occurs in a particular setting to identify the most appropriate channels, which is likely to include the use of social media to reach large numbers of people, was recognized as necessary to ensure effective, clear, well-coordinated communication during health crises [42, 43]. Guidelines highlighted the importance of the following in terms of risk communication. First, the local context and the channels commonly used locally to allow smooth communication with the community. Second, the local social norms, beliefs, perceptions/cultures that might inform the message framing and its delivery. Third, the socio-economic (e.g. poverty) and political (e.g. conflict or peace) context that influence the message and its delivery [35, 37, 38].

Some global guideline documents also explicitly recognize the need for a deep contextual understanding of local beliefs, needs and perceptions to inform the content (as well as mode of delivery) for effective communication about risk in times of disaster [26, 44]. They describe how the failure to learn lessons on the importance of taking local contexts into account from the West Africa Ebola outbreak led to a higher case fatality rate (CFR) in the subsequent DRC outbreak (which had a CFR of 67.8% compared with 36.7% in West Africa). They also note that poverty and volatile political situations can make many population groups significantly more vulnerable, making effective communication more challenging, but very necessary. Understanding and then following locally acceptable models of service delivery and engagement in terms of context, culture and social norms and avoiding using technical and difficult medical language that people do not understand that can lead to suspicion is highlighted by several documents [20, 42, 44–48].

There is less attention to contexts of other community engagement activities, despite a professed commitment to reflecting on lessons from past responses to improve community-centred approaches. Stakeholders recognise that most policies do not consider involvement of community members to respond to health crises. Learning from past epidemics/pandemics, a WHO stakeholder meeting was held to discuss the gaps, needs, and future research topics for community-centred approaches in response to a health crisis. The discussion of COVID-19, as an example of epidemics, informed its emerging strategies and policies for community-centred approaches (including training and community partnerships for data-generation and use) [41]. That means responders should partner with the community to train them to effectively generate and use data [40].

### Mechanisms of engagement

In total, five key clusters of mechanisms by which community engagement is conducted emerged in our review (Fig. 2) and the focus was different across the two types of documents included. In the conceptual and framework literature (Fig. 2), there are actions relating to: mapping and networking of frontline affected communities and responders, participatory approaches to engage stakeholders and community members and actions to promote coordination and communication were all highlighted. The global guideline documents (Table 1) mainly highlighted the incorporation of community engagement in policy and training guidelines and use of two key communication channels: community focal persons and social media.

#### Identify existing community stakeholders and networks for engagement

Lessons learned from the West Africa epidemic (not implemented in DRC) underline the need to rapidly identify and work closely with local frontline responders and communities [1]. This involves the chiefs, herbalists, youth leaders, traditional health attendants, community health workers, teachers and others. A wide reach of involved stakeholders helped to promote locally acceptable beliefs, such as treatment and burial practices, including offering as-safe-as-possible options for home care where access to care centres is not possible [1, 4].

The key to rapid inclusion of a wide range of community leaders is the use of existing community structures [18, 25, 35, 48]. Existing community structures (local/traditional councils, local health facility, health facility committees, development groups, women’s groups, etc.) can immediately respond to crises while seeking external help and they can provide human and other resources, mobilising the entire community. Importantly, they can prepare the community when there are warning signs of disaster. Involving the community from the start improves acceptance of external responders. These existing community structures should be identified and funded by governments and international stakeholders. The implementing partners (responders) should utilize and work with existing groups and networks for crisis response actions and ensure feedback loops so that community level experiences and insights can inform policy [18, 25, 35].

#### Participatory approaches to engagement

Many authors note the importance of collaborative action involving formal authorities and citizens, involving community members at all levels of the response to increase their coping capability and develop trust by taking public opinion into account during response actions [18, 25, 35]. Giving tasks and responsibility to community members, through co-production and partnership, to help in responding to the health crisis is an effective way to help them own the response actions. For instance, involving community members in tracking rumours and in developing mitigation strategies to address them, means they are more likely to be effectively tackled [18, 25, 35, 44].

Citizen science is a co-production mechanism that has been demonstrated by researchers, especially in the agricultural field, to be particularly effective [43, 45]. Workshops and other activities bring community members and scientists together to discuss and co-design research and interventions, considering the needs and expectations of communities. This benefits both the science/emergency response community through knowledge generation and secondly, benefits the affected communities through more directly meeting their needs and empowering them [41, 43, 45]. Participatory research can enable marginalised groups in making their own decisions [49]. This process can inform implementation of health programme in preventing or responding to health crises.

However, participatory approaches can be difficult to do well, and need careful implementation by skilled professionals and close monitoring. Some studies found that sub-dividing tasks to allocate to individual participants or small groups was helpful. This helps to produce an aggregate effect at the community level [50, 51].

Training was often regarded as an important mechanism for building the capacities both of community members and healthcare and other emergency-response professionals [40, 41, 44]. For community members, training focused on their contributions to responding to specific types of crises, for example training on what they can do to monitor infectious disease, support surveillance and perform contact tracing. Training for professional responses included competency-based training for public health emergency response, training in disaster preparedness and management and training in effective collaboration with community leaders. Studies noted the importance of involving people from different backgrounds including both healthcare professionals (doctors, nurses) and other professionals (with social, environment, development background, etc.). Furthermore, benefits of partnerships for training between academics and community-based organisations were noted, as well as coalitions for media-emergency operation centres [48, 52].

#### Coordination, risk communication, and community dialogue

Effective coordination between actors is critical because poor information sharing and coordination can undermine collective decision making and actions [27, 34]. Clear, synchronised risk communication can be valuable to inform affected populations who are the first responders in a crisis, and can help to build trust in the response [28, 32]. This is essential to enable a true dialogue between health and response authorities and local citizens [32].

The coordination of crisis management across these different stakeholders is often difficult, as experience from the Office of Emergency Preparedness (OEP) in Netherlands attests [28]. It recommended the development of proper technology to facilitate easy and rapid action including new technologies for communication of disaster information and reinforce partnership between actors. Furthermore, the creation of guidelines on community engagement (CE) with specific health service related experts, and coordination among a range of different groups, including funders, scientific experts, manufacturers, researchers, vaccine logistic agencies and regulatory authorities, is recommended [34, 40].

Many papers note the importance of having risk communication plans and protocols, including the use of social media and digital technologies, to facilitate coordination of the response between different actors [36–38, 44]. Others also underline the need for citizens to be involved in developing those plans to enable mobilization of community-led and collective action to support emergency response actions [18, 25, 35].

In the Manoncourt et al. book of 2022, the importance of learning from the mistakes of the responses to the Ebola epidemics in West Africa and the DRC was underlined [26]. The risk communication there was focused on what to do and what was forbidden (on infection control grounds). Communication protocols did not consider the individual and collective behaviour that facilitated the spread of the disease and the expectations of the local populations (particularly around dignified burial and care for the sick). This was a source of challenge and local resistance until community engagement improved when local leaders were brought on board [26].

#### Response guidance and training strategies

Taking up the call from some of the framework papers, to develop policy guidance and training on community engagement, a number of global guideline documents echo the key strategies that are needed [41, 44]. These include the following: First, the training of local actors for a rapid response to outbreaks. Second, partnership between stakeholders involved in public health emergencies (public and private) is key. Third, use of community-generated data and knowledge through participatory approaches. Fourth, take feedback from the community to adapt interventions and make or change the policies. Fifth, work directly with communities to understand their needs and engage them in disease risk assessment and use digital applications to develop alert mechanisms. Sixth, incorporation of community engagement into preparedness, readiness and response plans. Seventh, training for health experts working with the community in public health, in communication skills.

Specifically in relation to communication, community focal points, use of social media and access to internet during crises were all noted as important for keeping close contact with the community [26]. Some global guideline documents go into considerable detail of how to implement effective engagement strategies. For example, GOAL GLOBAL [20] outlines several strategies for improving effectiveness of community engagement. These include defining your ‘community’ and identifying smaller sub-groups (or ‘units’) that are easier to work with. These sub-groups should be defined by common and shared characteristics or amenities (e.g. public latrines, shared water points, markets, commonly used shops and churches) so that they are a cohesive ‘community’, and given an identifying name that is commonly known in the area. Each sub-group will have a focal point person and all the response actions should be planned and executed together with them and should be led by their availability [20].

#### Channels of communication

The development of technology has given people access to a wide range of information through social media and is considered in a range of global guideline documents. These particularly recommend the following two communication strategies. First, undertaking structured dialogue with communities through appointed focus people in each community [35, 37]. Second, judicious use of social media/digital technology, including tailored messages for different groups [35, 37, 38]. Social media provides an accessible platform for information sharing about the crisis. It is a channel where people also share their own experience so an effective crisis communication plan is important to monitor and counter misinformation. Used wisely, social media for crisis communication can have a high influence on people’s behaviour, such as physical distancing, mask-wearing and vaccine uptake during COVID-19 [43, 46].

Developing targeted messages for specific social groups is recognized as an asset and is often necessary to reach marginalised groups [43]. Social media provides an opportunity to use two-way communication by responding to questions and asking for feedback. Documents noted the need for social media messages to be clear, action-oriented, easy to understand and to share and conversational in style. They noted the need to know who the target audience is and what their messaging preference is as well as which social media channels are commonly used [22, 26, 41, 43]. Using the communicator that is most trusted by the target audience is key. For instance, during the COVID-19 pandemic, partnerships were developed with diaspora influencers, and community-based organizations to better meet the needs of the target audience. Audience segmentation was applied by spokespersons to better deliver the messages [42–48].

### Assessing the outcomes

There are currently no agreed indicators on successful outcomes for community involvement in crisis response, and studies reported on a range of process and proxy indicators and direct health outcomes from both theoretical/conceptual literature and grey literature (guidance documents). These included the following. First, **social resilience developed:** capacity to absorb and resist to shocks and capacity to adapt to the difficult situation. Second, **sustainable structures developed:** reliable existing structures ready for any emergency, valued and connected people and adapted channels of communication. Third, **health indicators improved:** overall health services improvement, preventable deaths avoided and decrease of disease prevalence. Fourth, **trust built:** sustainable partnerships created and responsibilities (tasks) given to community members who are involved in the decision-making process. Fifth, **community development supported:** social capital identified, ownership spirit created and rapid response is possible. Sixth, **programme development enabled:** effective communication, measurable influence of communities on services & policy/ delivery/ access, local groups created ready for action. Collectively, these lead to increased capacities of the health sector and other officials to mobilise and use community resources; communities receive clear information and get answers to their concerns; more effective response (e.g. rapid reduction of spread; lower CFRs); communities able to propose and implement response activities; communities to cooperate and adhere to agreed response activities (e.g. contact tracing, quarantine); and communities take responsibility for sustaining their responses (e.g. to environmental protection) and show pride in and ownership of their response.

#### Building social resilience capacities

Several conceptual papers address the notion of social resilience (the ultimate outcome), describing the three main categories: ability of health and social systems to absorb and resist a shock (to ‘weather the storm’ before picking up their previous functions again), to adapt to the shock and continue to function while doing so and to transform as a result of the shock and be stronger and better prepared for the future [36–38].

Moya and Goenechea (2022) also differentiate between individual capacities to adapt to change and wider social structures’ ability to respond to change and build resilience of individuals within them [53]. Although the authors admit that there is a need for new models of social resilience, they and others note how actively involving communities in crisis response can significantly contribute to building resilience at community – as well as individual – levels [31, 33, 46, 47]. For example, partnerships between academic institutions and community-based organizations, through citizen science and other mechanisms, have had a demonstrable effect on enhancing community resilience by increasing knowledge and ownership of response actions, trust in local leaders, collaboration and mutual support [48, 51, 52]. Training in particular skills – from surveillance and monitoring through to networking and communication – can contribute to building individual, social and informational capital [50, 52, 53].

#### Development of sustainable structures

Global guideline documents typically did not detail expected outcomes and appeared to be working on the a priori assumption that community engagement per se is an intermediate outcome that can potentially facilitate health and developmental outcomes. While this may be true, it would nevertheless be pragmatic to be clear what expected outcomes each mechanism is intended to lead to, to avoid inappropriate or unclear actions and avoid a tick-box approach to doing community engagement. It has been shown that using the existing community structures, such as health facilities, existing social groups and local community-based organisations, reinforce ownership spirit and preparedness leading to the community members being first responders [1, 18, 19, 21, 26, 28]. This helps in making the response actions more sustainable [16, 19, 26] and the community becomes ready for future crises [1, 25, 26, 30]. Ultimately, participation in the processes of decision making can increase sustainability because it avoids creating dependencies on outsiders to keep offering benefits [49]. Specifically, participation is seen as leading to increased effectiveness and efficiency of mutually agreed response actions, empowerment of marginalized social groups (if inclusion is broad), and improved sustainability of developed solutions through increased social capital and commitment [35, 49, 51, 52].

##### Improvement of health indicators

The ultimate outcome of interest for health organisations (such as theWHO) is an improvement in health indicators. Effective communication (e.g. from community focal points) and engagement with a wide range of stakeholders at different response levels was recognised as key to contributing changes in health indicators [26, 44, 45, 48]. The precise mechanisms by which these improvements are achieved is not detailed; although good, permanent and effective communication about risk is seen as a key element to keep the population healthy in time of crisis and avoid preventable deaths [19, 20, 22, 25, 26, 30]. This helps to reduce the morbidity and the CFR when the community members are aware of what to do, how, and where to go in case evacuation is needed [16, 30–32, 35–37].

##### Building trust

Many documents also explicitly noted the need for community engagement activities to build trust as a key outcome that underpins effective emergency responses [20, 44, 46]. Trust was also seen as important for building acceptance and good reputation (of responders) and community ownership [20, 41, 44]. The aim of sustaining actions and resources supporting communities to respond to shocks was also noted, [42, 45, 48] though few documents discussed the need for long-term investments to achieve this [41]. Trusted communication and two-way dialogue between responders and communities was seen as key to building good relationships that lead to more effective, sustainable responses [33–35]. Good communication and trusted relationships then lead the way to effective participation between responders and communities [28–30].

##### Community development

Studies have explicitly shown that empowering local community members creates ownership spirit and triggers the community feeling responsible for the response at the first instance [1, 3, 16, 19, 26]. Communities also need support from outside responders working jointly as partners for a rapid response. This enables the development of social capital and community capacities to respond at the front line [1, 3, 16, 19, 26]. Participation can help to create a sense of ownership of the response actions, providing it is an inclusive and dynamic process and works with existing structures where possible [18, 48, 49]. For example, a community can contribute to response mechanisms by bringing new ideas that can inform future response plans [48, 50, 52, 53]. In many countries this has taken the form of communities helping to track rumours and then contributing to shaping the mitigation strategies to address them in response to health shocks [18].

##### Programme development

In times of crisis, there is a need to have effective and consistent communication with the community members to ensure that the community get the right information at the right moment [1, 18, 25, 30, 37, 38]. This helps to reinforce the relationship between responders and community members. Understanding the channels of communication commonly used locally is another key element of success to ensure the message reaches people who need it the most and by the trusted spokespersons [18, 30, 37, 38]. The local means of communication can be further developed to respond to the standard of technology in collaboration with the community members or their representatives (25, 29, 37, 38).

## Discussion

Our realist-informed scoping review analysis sought to explore how community engagement in crisis response has been conceptualized and what provisions have been made in guidelines and policy documents to enable it (sections 1 and 2). It showed that the extent of information available about the contexts, mechanisms, and outcomes of community engagement and, most importantly, of how these CMO categories interact to shape effective community engagement, is not very extensive. Context issues are important because they shape the nature and implementation of community engagement mechanisms, but they are often poorly described. There is growing evidence of promising mechanisms of community engagement though a much better understanding is required of how they are shaped by particular contexts, and how and why they can lead to particular outcomes. As a result of a poor understanding of what outcomes are achieved by what mechanisms in what contexts, the outcomes of community engagement are frequently not well-defined. Hence, there are no globally agreed indicators.

Overall, we found that a full understanding of the interactions between context (crisis settings, weak health systems capacity, specific cultural contexts etc.) and mechanisms (modes of community engagement and participation in decision making and response actions) to achieve particular outcomes (including building trust, engagement in response actions, supporting social capital, ownership and sustainable capacities to respond to future crises) is often lacking in both the scientific conceptual literature and, particularly, in global guideline documents. Yet without such understanding, effective strategies that can build resilience of communities to respond to shocks cannot be developed and implemented. Only 7 of the 26 reviewed documents (as seen in Table 1) were able to provide any degree of description of the interconnections between the contexts, mechanisms and outcomes that they detailed and only three of these (Table 1) provided a high level of detail.

These findings may reflect the tendency of both conceptual literature and official guidelines to provide generic information rather than case-specific detail, though the strongest documents were able to distil generic principles and approaches from grounded and worked examples (that illustrated CMO interactions). Nevertheless, with limited analytical evidence of CMO interconnections, we cannot assess what outcomes are achieved by what mechanisms in which contexts. The outcomes of community engagement are frequently ill-defined, so there are no globally agreed measures and indicators. Consequently, it is difficult to assess what really works and therefore difficult to inform impactful community engagement strategies. To answer these questions, further empirical research is needed.

Figure 2 provides a framework to inform policy and future research by summarising key components of CMO categories that we identified inductively from existing guideline and framework documents according to the context, mechanisms and outcomes relating to community engagement. The figure 2 must be here please. 

This study has shown that constant communication with community members in time of crisis and using the channels that community is most familiar with helps to bring them on board in the response and reduce their level of vulnerability [26, 27, 34]. This is an important element that policy makers should consider while making new policy or changing policy. Policy at any level should be informed by the community needs for its smooth implementation and effectiveness.

**Fig. 3:**
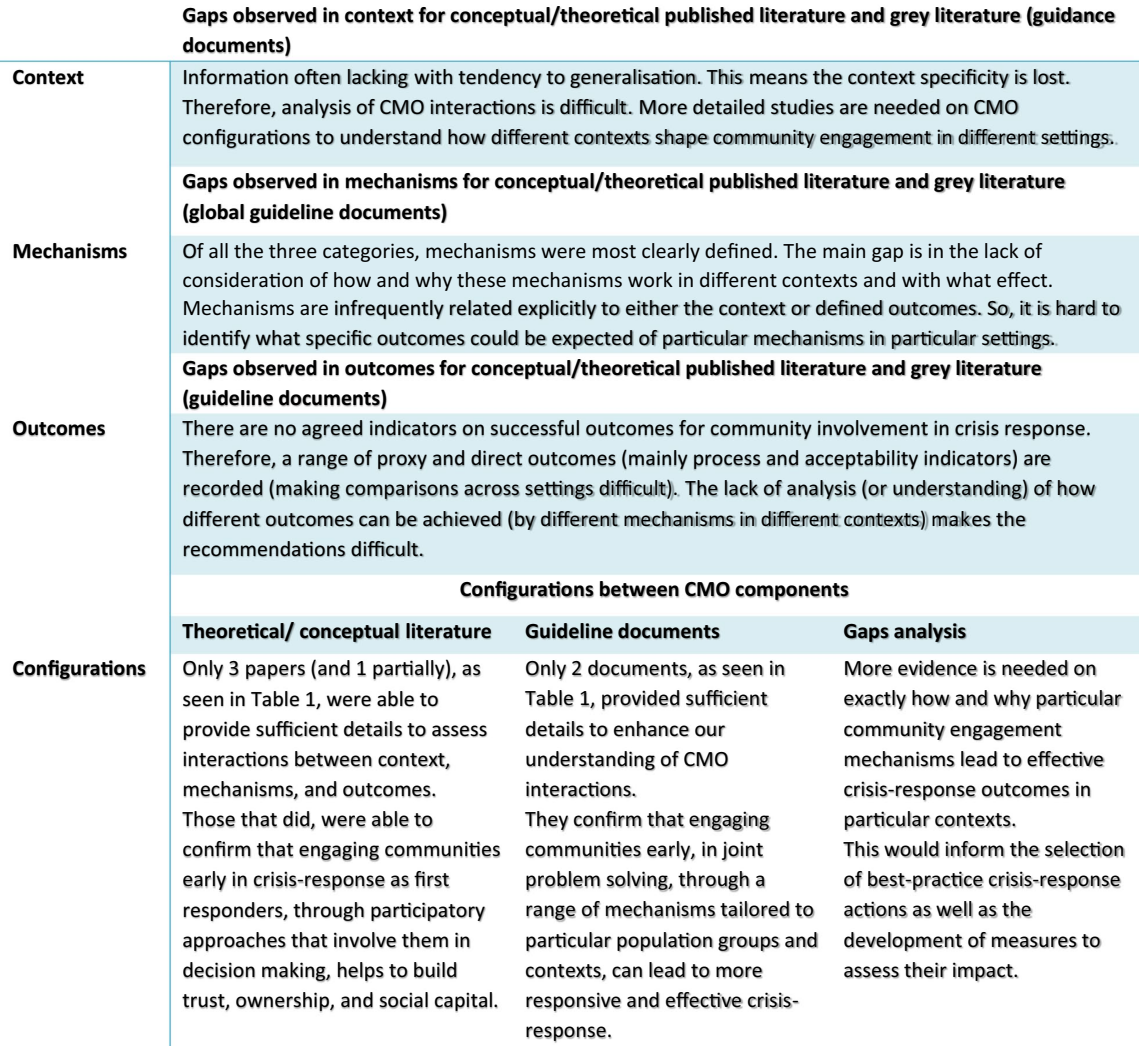
Summary of CMO (context, mechanisms, outcomes) gap analysis and configurations for community engagement in health crisis response

In addition, Fig. 3 below summarises the possible configuration between context, mechanisms and outcomes from our analysis and it also informs researchers by identifying key gaps that further research needs to address.

## Conclusions

This study has provided the first systematic attempt to examine theoretical literature and formal guideline documents on community engagement in crisis response using a systematic realist-informed approach to identify existing knowledge of the contexts and mechanisms that can lead to particular outcomes while responding to health crises. We found that available evidence on each of the CMO categories of context, mechanism and outcome was promising, but few documents provided detail on all three and even fewer were able to show evidence of the interactions between them. Without a good understanding of why specific community engagement mechanisms can achieve positive outcomes for crisis response, in particular contexts and for particular population groups, we cannot properly inform effective community engagement strategies. Mapping local stakeholders and vulnerable groups in communities was found to be an important first step in engaging communities for crisis preparedness and rapid response. This paper shows that engaging community members at all steps of the response, including at the planning level, helps to build the trust that leads to effective implementation of response actions. It shows that community members can be effective first responders when they are empowered and prepared for a crisis, underlining the fact that social capital does exist in communities. This paper is a contributing step to a better understanding of which community engagement mechanisms can work in which settings, but more evidence is needed on exactly how and why particular community engagement mechanisms lead to effective crisis response outcomes in particular contexts and for particular population groups. This would inform the selection and testing of best-practice crisis response actions in community engagement as well as the development of measures to assess their impact. It is better to understand the community needs before any crisis response and policy-making process that should be informed by feedback from community members.

## Data Availability

Data will be accessible as open access and we would like to keep CC BY copy right. Data will be reposited in the repository of LSHTM after the manuscript is accepted for publication and a link to access them will be provided by then.
